# Determinants of maternal knowledge on neonatal danger signs and care-seeking practices in a rural area of southeastern Ethiopia

**DOI:** 10.1093/inthealth/ihab084

**Published:** 2021-12-17

**Authors:** Tamirat Getachew, Tesfaye Assebe Yadeta, Teklehaimanot Gereziher, Addis Eyeberu, Merga Dheresa

**Affiliations:** School of Nursing and Midwifery, College of Health and Medical Sciences, Haramaya University, P.O. BOX 138 Dire Dawa, Harar, Ethiopia; School of Nursing and Midwifery, College of Health and Medical Sciences, Haramaya University, P.O. BOX 138 Dire Dawa, Harar, Ethiopia; Nursing Department, College of Health Science, Aksum University, P.O. BOX 158 Aksum, Northern Ethiopia; School of Nursing and Midwifery, College of Health and Medical Sciences, Haramaya University, P.O. BOX 138 Dire Dawa, Harar, Ethiopia; School of Nursing and Midwifery, College of Health and Medical Sciences, Haramaya University, P.O. BOX 138 Dire Dawa, Harar, Ethiopia

**Keywords:** client satisfaction, delivery service, healthcare, mothers

## Abstract

**Background:**

Most infants in southeastern Ethiopia are either born at home or discharged from the health facility early and families should be able to recognize signs of newborn illnesses and bring the sick newborn to a health facility to receive care. However, studies are limited and the available studies were conducted in urban areas and/or at an institution level. This study aimed to assess the determinants of maternal knowledge of neonatal danger signs and care-seeking practices.

**Methods:**

A community-based cross-sectional study was conducted on 520 post-natal mothers using a multistage sampling method from 1 to 30 March 2019. The data were analysed using SPSS version 20 using binary logistic regression. Statistical significance was declared at p<0.05.

**Results:**

Mothers’ level of knowledge of neonatal danger signs was 50.2% (95% confidence interval [CI] 46.3 to 54.3) and 61% of them sought healthcare when they noticed danger signs. Maternal education level (adjusted odds ratio [AOR] 2.15 [95% CI 1.11 to 4.17]), husband's education level (AOR 2.05 [95% CI 1.07 to 3.94]), residency (AOR 5.83 [95% CI 2.77 to 12.24]), antenatal visits (AOR 2.10 [95% CI 1.13 to 3.90]), antenatal care (ANC) counselling (AOR 4.33 [95% CI 1.88 to 9.98]) and knowledge about essential newborn care (AOR 3.91 [95% CI 2.05 to 7.48]) were the determining factors.

**Conclusion:**

The mothers’ level of knowledge of neonatal danger signs was low and unsafe care-seeking practices were identified. The mothers’ education level, husbands’ education level, residence, ANC visits, counselling during ANC and knowledge about essential newborn care were found to be statistically significant determinants. Most of the mothers take their sick neonates to traditional healers and provide home remedies. Intervention modalities focusing on maternal counselling on the most common symptoms of illness in neonates are essential to increase mothers’ recognition of illness and improve care-seeking practices.

## Introduction

Danger signs are signs that can be easily identified by non-clinical personnel, including mothers and caregivers.^1^ Neonatal danger signs are clinical signs that indicate a high risk of neonatal morbidity and mortality and are needed for early therapeutic intervention. Early detection of neonatal illness by identifying neonatal danger signs is an important step towards improving newborn survival.^1,2^

Globally, approximately 4 million babies die in the first 28 d of life. In 2019 an estimated 2.4 million neonates died in their first month of life.^3^ A neonatal mortality rate of 30/1000 babies was recorded in Ethiopia in 2019 (120 000 babies die every year in the first 4 weeks of life).^4^ That is, 1 in every 35 neonates dies within the first month nationally and 37/1000 die in the Oromia region. In Ethiopia, 62% of live births have high mortality risks that are avoidable and treatable if mothers seek healthcare for their neonates.^5–7^

Knowledge about the severity of neonatal illness (i.e. knowing when to act) and appropriate lifesaving actions is essential to prevent or avoid health problems. Thus, improving maternal knowledge of the signs of neonatal illness significantly decreases neonatal morbidity and mortality.^8,9^ A lack of specificity of the clinical manifestations of various neonatal illnesses, resulting in difficulty in making an early diagnosis and a delay in seeking care, resulting in high mortality, are some of the explanations for newborn health problems.^10^

Studies conducted in Ethiopia have shown that mothers have poor knowledge of neonatal danger signs. Mothers’ practices in treating sick neonates are unsafe, as most mothers take their sick neonates to traditional healers and provide home remedies. Most neonatal deaths occur at home, indicating a lack of early recognition of danger signs and low treatment-seeking practices of mothers (caretakers) from modern healthcare services.^11,12^

Even though the Ethiopian government has increased the provision of quality community-based newborn care services, including management of newborn sepsis, and has strengthened supportive systems with a focus on woreda (administrative district) capacity building, the change in neonatal mortality is not as significant as the change in post-neonatal and child mortality.^5^

Although mothers’ knowledge of neonatal danger signs plays a critical role in reducing neonatal morbidity and mortality, studies seem to be limited. Since the available studies were conducted in urban areas or at an institution level,^13^ they do not address the rural community, where knowledge of neonatal danger signs is relatively low and mothers have home delivery. Previous studies assessed knowledge from post-natal mothers who delivered 2 y ago,^14^ which may result in recall bias and failure to differentiate between the neonatal and post-neonatal periods. Therefore the level of maternal knowledge might be misreported. This study aimed to assess the determinants of maternal knowledge of neonatal danger signs and care-seeking practices in southeastern Ethiopia from 1 to 30 March 2019.

## Methods

### Study setting and period

The study was conducted from 1 to 30 March 2019 in the Chole woreda, which is found in the southeastern part of Ethiopia. The woreda is about 291 km away from Addis Ababa, the capital of Ethiopia. Chole woreda has a total of 20 kebeles (4 urban and 16 rural; a kebele is the smallest administrative unit in Ethiopia). The woreda has a total population of 120 764, of which 61 568 are males and 59 196 are females, with 4191 annual live births.

According to the 2017 report of the Chole health bureau, the estimated number of women of childbearing age was 21 738 (7386 urban and 14 352 rural).^15^ There were 2095 (712 urban and 1383 rural) mothers who had live births in the past 6 months in the woreda. The woreda has 4 health centres, 18 health posts, 10 private clinics and 8 drug stores. According to the 2017 report, the health coverage of the woreda reached 64.56% in 2016.

### Study design and population

A community-based cross-sectional study design was applied using a descriptive quantitative method. All mothers who gave birth within the 6 months before the study period and resident for at least 6 months in the Chole woreda were included in the study. Mothers who gave birth in the past 6 months but were unable to communicate because of serious illness or impaired cognition during the data collection period were excluded from the study.

### Sample size determination and sampling procedure

The sample size was calculated using a single population proportion formula with the following assumptions: 95% confidence level 1.96, margin of error (d) 0.05, design effect 1.5; a reasonable proportion (which gave the largest sample size) of mothers’ knowledge of neonatal danger signs (p=0.313) was taken from a previous study conducted in Wolkite Town, Ethiopia in 2017^11^ and adding a 5% non-response rate. Thus the final sample size was 520.

A multistage sampling method was employed to select the study subjects. The Chole woreda has 16 rural and 4 urban kebeles. First, kebeles were stratified into urban and rural kebeles and five rural kebeles and two urban kebeles were selected randomly by assuming that kebeles in urban areas were homogeneous and kebeles in rural areas were also homogeneous. The sample size was distributed to the seven kebeles proportionate to the size of their population. Finally, the subjects who had been included in the study from each kebele were identified by using a simple random sampling technique (computer-based) based on the sampling frame obtained from the kebele health extension workers’ registration books (N=2095). The selected households were located with the help of kebele health extension workers and administrators of the kebeles. For a household with more than one mother who gave birth in the past 6 months, one of the mothers was selected using a lottery method.

### Data collection methods

Data were collected using an interviewer-administered structured questionnaire that was adopted from the Safe Motherhood questionnaire developed by the Maternal and Neonatal Health Program of the Johns Hopkins Program for International Education in Gynecology and Obstetrics.^16^ The data were collected by training seven diploma nurses and supervised by three nurses (with bachelor of science degrees) who were fluent in the local languages Afan Oromo and Amharic. The reason nurses were chosen is because, at the end of the data collection, health education was provided to the respondents with poor knowledge of neonatal danger signs. A brief introductory orientation was given to the study participants by data collectors regarding the purpose of the study. An explanation was given about the importance of their involvement, then mothers who volunteered were interviewed face to face using structured and pretested questionnaires at the household level.

### Operational definition and measurement

Key danger signs are those signs that affect the survival chances of a neonate and demand immediate medical care. These includes poor/not sucking, fever/high body temperature or being hot as perceived and reported by mothers, hypothermia, convulsion, increased respiratory rate/fast breathing, vomiting, chest retraction/in drawing, jaundice (yellow soles, palms and sclera), lethargy and umbilical redness or draining pus/sign of infection.^17,18^

Knowledge was defined as the state of awareness of mothers of key neonatal danger signs.1 Knowledge was measured by the mother's ability to mention the 10 World Health Organization (WHO)-listed neonatal danger signs without prompts from the interviewer. Based on the classifications of many scholars and researchers,^11,19-21^ the knowledge level was categorized into good knowledge and poor knowledge according to the respondent's ability to mention 3 of 10 WHO-listed neonatal danger signs. Mothers who were capable of mentioning three or more key WHO-identified danger signs for neonates were classified as having good knowledge, while mothers who were able to mention two or fewer key WHO-identified neonatal danger signs were classified as having poor knowledge.^11,19–21^

Neonatal danger signs are symptoms that complicate the life of the neonate and occur during the neonatal period (during the first 28 d of life).^22,23^

Those mothers who took their neonates to the hospital or health centre immediately after the neonate developed any danger signs were considered to have good care-seeking practices, otherwise they were considered as having poor practices.^21^

### Data quality control

The questionnaire was first prepared in the English language and translated to the Amharic and Afan Oromo languages, which are used for communication in the local community, then back to English by different language experts to check the consistency of the data. The questionnaire was pretested on post-natal women in the Guna district on 5% of the total sample size before the beginning of the actual data collection period. Findings and experiences from the pretest were used in modifying and reshaping the research data collection tools. Before data collection, training was provided to data collectors and supervisors about the objective of the study, confidentiality of information, respondent's rights, privacy and techniques of the interview. The completeness of the questionnaire was checked by the principal investigator and supervisors daily. Double data entry was done by two data clerks and the consistency of entered data was cross-checked by comparing two separately entered data files into EpiData (http://www.epidata.dk).

### Data processing and analysis

The data were coded, entered and cleaned in EpiData version 3.1 and then were exported to SPSS for Windows version 20 (IBM, Armonk, NY, USA) for analysis. A descriptive statistical analysis was employed to describe the characteristics of participants. For analysis of the outcome variable, good knowledge was coded as 1 and poor knowledge was coded as 0. The information was presented using frequencies, tables and figures. Multicollinearity was checked using the variance inflation factor (VIF) and standard error (SE) and variables with a SE >2 or VIF >10 were dropped. The goodness of fit was tested by the Hosmer–Lemeshow statistic and omnibus test. The model was considered a good fit since it was found to be insignificant for the Hosmer–Lemeshow statistic (p=0.648) and significant for the omnibus test (p≤0.001). Bivariate and multivariate analyses were used to observe the association between each independent variable and the outcome variable by using binary logistic regression. All variables with a p-value ≤0.25 in the bivariate analysis were included in the final model of multivariate analysis to control all possible confounders. The direction and strength of the statistical association were measured by the odds ratio (OR) with 95% confidence interval (CI). Adjusted ORs (AORs) along with 95% CIs were estimated to identify associated factors with knowledge about neonatal danger signs by using multivariate analysis in the binary logistic regression. Finally, statistical significance was declared at p<0.05.

### Ethical considerations

Ethical clearance to conduct this study was obtained from Haramaya University, College of Health and Medical Sciences, Institutional Health Research Ethics Review Committee before starting the data collection process. An official letter to conduct a study was also obtained from the woreda and kebele government officials as needed.

Informed, voluntary, written and signed consent was obtained from each study participant before the interview after explaining the purpose of the study and their right to refuse or discontinue the interview at any time. The consent was written in the local language (Amharic and Afan Oromo). The interviewer read the consent form to respondents if they were unable to read it themselves. There was an option to put their fingerprint if they were unable to sign their name. They were also informed that the information obtained from them would be treated with complete confidentiality (respondent's name and other identification were not on the questionnaire) and would have minimal risk to them. All methods used throughout the study were carried out per Haramaya University guidelines and regulations.

## Results

### Sociodemographic characteristics

A total of 510 of 520 mothers of babies up to 6 months of age were recruited, yielding a response rate of 98.1%. The mean age of the participants was 28.48 y (standard deviation 4.68) and the minimum age of the mother at the time of the interview was 19 y. The majority of the mothers were married (467 [91.6%]) and rural residents (333 [65.3%]). A total of 337 (72.7%) of them were Orthodox Christian and 255 (50%) belonged to the Amhara ethnic group (Table 1).

**Table 1.** Sociodemographic characteristics of study participants who delivered in the past 6 months in southeastern Ethiopia, 2019 (N=510)

Variable	Category	Frequency	Percentage
Sex of child	Male	269	52.7
	Female	241	47.3
Mother's age (years)	15–24	105	20.6
	25–34	340	66.7
	35–44	65	12.7
Mother's education level	Primary and below	178	34.9
	Secondary and above	332	65.1
Marital status	Single	9	1.8
	Married	467	91.6
	Divorce/separated	27	5.3
	Widowed	7	1.4
Father's education level (n=467)	Primary and below	273	58.5
	Secondary and above	194	41.5
Religion	Orthodox	371	72.7
	Muslim	114	22.4
	Protestant	25	4.9
Ethnicity	Oromo	253	49.6
	Amhara	255	50.0
	Tigre	2	.4
Residence	Urban	177	34.7
	Rural	333	65.3
Occupation	Government employee	22	4.3
	Private employee	28	5.5
	Housewife	353	69.2
	Merchant	29	5.7
	Farmer	67	13.1
	Other^a^	11	2.2

a Student and daily labourer.

### Maternal health services and obstetric conditions

A total of 363 (71.2%) of the mothers were multiparous (mothers who had two or more childbirths) and 63 (87.6%) of them had at least one abortion. Among the 483 mothers who had antenatal care (ANC), 237 (49.1%) had four and more visits. The majority of respondents (392 [76.9%]) were seen by a midwife/nurse. Only 364 (75.4%) respondents attended regular ANC counselling and counselling on neonatal danger signs was the least covered area of counselling during ANC (in 21 [5.8%] respondents) (Table 2).

**Table 2.** Maternal health-related factors among mothers who gave birth in the past 6 months in southeastern Ethiopia, 2019 (N=510)

Variable	Category	n (%)
Number of pregnancies	1	112 (22.0)
	2–4	363 (71.2)
	≥5	35 (6.9)
Abortion	Have abortion history	63 (12.4)
	No abortion history	447 (87.6)
ANC	Yes	483 (94.7)
	No	27 (5.3)
ANC visits (n=483)	≤3	246 (50.9)
	≥4	237 (49.1)
ANC provider (n=483)	Doctor/health officer	7 (6.7)
	Midwife/nurse	392 (76.9)
	HEW	84 (16.4)
Counselled during ANC (n=483)	Yes	364 (75.4)
	No	119 (24.6)
Counselled during ANC (n=483)	Family planning	170 (46.7)
	Maternal nutrition	143 (39.3)
	Breastfeeding	136 (37.4)
	Hygiene	106 (29.1)
	Immunization	98 (26.9)
	HIV awareness	90 (24.7)
	Neonatal danger signs	21 (5.8)

HEW: health extension worker; HIV: human immunodeficiency virus.

### Obstetric conditions

The majority of mothers (376 [73.7%]) delivered at health institutions. A total of 381 (74.7%) of the respondents had immediate post-natal care (PNC; the care given to post-natal mothers within the first 24 h after delivery) and the majority (325 [85.3%]) of them were assisted by midwives/nurses. However, only 138 (36.2%) of the respondents were counselled on neonatal danger signs during PNC (Table 3).

**Table 3.** Obstetric conditions among mothers who gave birth in the past 6 months in southeastern Ethiopia, 2019 (N=510)

Variable	Category	n (%)
Place of delivery	Health institution	376 (73.7)
	Home	134 (26.3)
Delivery assistant	Health professional	376 (73.7)
	TBA	54 (10.6)
	TTBA	68 (13.3)
	Relative (friend)	12 (2.4)
Mode of delivery	SVD	487 (95.5)
	Instrumental delivery	15 (2.9)
	Caesarean section	8 (1.6)
Immediate PNC	Yes	381 (74.7)
	No	129 (25.3)
Caregiver during PNC (n=381)	Midwife/nurse	325 (85.3)
	HEW	47 (12.3)
	Other^a^	9 (2.4)
Counselled during PNC (n=381)	Yes	138 (36.2)
	No	243 (63.8)

a Physician or health officer.

TBA: traditional birth attendant; TTBA: trained traditional birth attendant.

### Source of information about neonatal danger signs

A total of 438 (85.9%) respondents had received information (heard) about neonatal danger signs, while 72 (14.1%) had no information (not heard) about neonatal danger signs. The most common (349 [68.4%]) source of information about neonatal danger signs was health professionals. Of the mothers who heard from the media, 38 (52.1%) and 35 (47.9%) of them got the information from television and radio, respectively.

### Knowledge about essential newborn care

Of 510 respondents, 291 (57.1%) had good knowledge (equal to or above the median score, ≥5), whereas 219 (42.9%) had poor knowledge (median score <5) about essential newborn care. Study participants were interviewed about knowledge of cord care, material to tie the cord, material applied to the cord, breastfeeding initiation time, exclusive breastfeeding, bathing time and immediate immunization. A total of 250 of the respondents stated string was used to tie the cord, 298 (58.4%) stated as nothing was applied to the cord, 386 (75.7%) stated that breastfeeding initiation time was within 1 h, 350 (68.6%) stated they exclusively breastfeed their newborn until 6 months and 301 (59%) bath the newborn after 24 h (Table 4).

**Table 4.** Knowledge about essential newborn care among mothers who delivered in the past 6 months in southeastern Ethiopia, 2019 (N=510)

Variable	Category	N (%)
Aware about cord care	Yes	378 (74.1)
	No	132 (25.9)
Material to tie cord (n=378)	Cord tie	250 (49.0)
	Unsterile material	80 (15.7)
	Don't know	48 (9.8)
Material applied on a cord	Nothing	298 (58.4)
	Antiseptic	34 (6.7)
	Butter	178 (34.9)
Breastfeeding initiation time	Within 1 h	386 (75.7)
	After 1 h	124 (24.3)
Exclusive breastfeeding until now	Yes	350 (68.6)
	No	160 (31.4)
Bathing time	Within 24 h	209 (41.0)
	After 24 h	301 (59.0)
Immediate immunization	Yes	27 (5.3)
	No	483 (94.7)

### Mother's knowledge of neonatal danger signs

**Figure 1.**
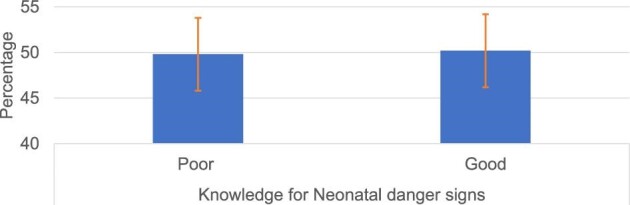
Total knowledge of neonatal danger signs among mothers who gave birth in the past 6 months in southeast Ethiopia, 2019 (n=510).

**Figure 2.**
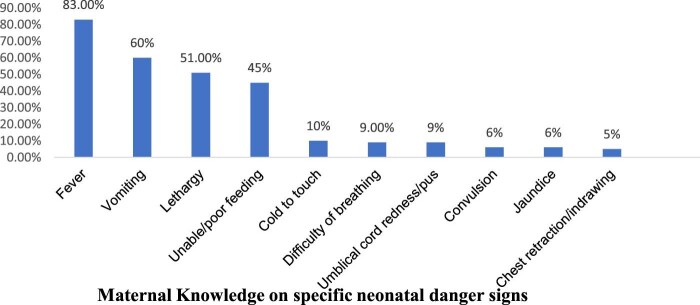
Maternal knowledge on specific neonatal danger signs among mothers who gave birth in the past 6 months, 2019 (n=510).

The majority (93.5%) of the participants were knowledgeable about at least 1 of the 10 WHO-recognized neonatal danger signs, but only 50.2% (95% CI 46.3 to 54.3) of the interviewed mothers were able to mention at least three neonatal danger signs (i.e. good knowledge) (Figure 1). The most frequently recognized neonatal danger sign, by 423 (82.9%) mothers, was hotness of the body (fever), followed by vomiting (306 [60%]) and lethargy (260 [51%]) (Figure 2).

### Care-seeking practice for neonatal danger signs

Of the 477 mothers who knew at least one neonatal danger sign, 292 (61.2%) perceived it as severe (the neonate might die) and they took their newborn to the nearby health facility, while 185 (38.8%) of them perceived it as not severe (those signs do not kill the neonate) and did not seek treatment at all. Regarding the place of seeking care, 51 (17.5%) of the mothers preferred to seek care for their sick neonate at home, 102 (35%) took the child to a health institution 139 (47.6%) took the child to a traditional healer.

The home treatments commonly practiced by the mothers were prepared from ‘damakesie’, garlic, ‘tenadam’ and honey. Moreover, participants used a mixture of lemon and ash to put on the neonate's head for management of tonsilitis, a match stick smock to control convulsions, tepid sponging for the relief of fever, exposure to sunlight for managing jaundice and rubbing oil on the cold body to avoid heat loss.

### Factors associated with knowledge of neonatal danger signs

The mother's age, mother's education level, husband's education level, residence, source of information, parity, number of ANC visits, counselling during ANC, place of delivery, counselling during PNC and mother's knowledge about essential newborn care were selected for the final model. After controlling for confounders in the multivariate model, mother's education level, husband's education level, place of residence, number of ANC visits, counselling during ANC and knowledge about essential newborn care were significantly associated with knowledge about neonatal danger signs. Mothers who attained a secondary or higher education level were 2.15 times (AOR 2.15 [95% CI 1.11 to 4.17]) more likely to have knowledge about neonatal danger signs compared with mothers who attained a primary or lower education level. Similarly, mothers whose husbands attained a secondary or higher education level (AOR 2.05 [95% CI 1.07 to 3.94)] were nearly twice as likely to mention at least three neonatal danger signs compared with husbands with a primary or lower education level. Mothers who live in urban areas were 5.83 times more likely to be knowledgeable compared with mothers who live in rural areas (AOR 5.83 [95% CI 2.77 to 12.24]) (Table 5).

**Table 5.** Factors associated with mothers’ knowledge about neonatal danger signs in southeastern Ethiopia, 2019 (N=510)

	Knowledge about neonatal danger signs, n (%)			
Variable	Poor	Good	COR (95% CI)	AOR (95% CI)
Age group (years)				
15–24	48 (45.7)	57 (54.3)	2.32 (1.22 to 4.41)*	1.01 (0.28 to 3.64)
25–34	163 (47.9)	177 (52.1)	2.12 (1.22 to 3.70)*	1.30 (0.46 to 3.64)
35–44	43 (66.2)	22 (33.8)	1	1
Maternal education level				
Primary and below	135 (75.8)	43 (24.2)	1	1
Secondary and above	119 (35.8)	213 (64.2)	5.62 (3.73 to 8.47) **	2.15 (1.11 to 4.17)*
Husband's education level				
Primary and below	163 (59.7%)	110 (40.3)	1	1
Secondary and above	60 (30.9)	134 (69.1)	3.31 (2.24 to 4.88)*	2.05 (1.07 to 3.94)*
Residence				
Rural	229 (68.8)	104 (31.2)	1	1
Urban	25 (14.1)	152 (85.9)	13.39 (8.27 to 21.69)**	5.83 (2.77 to 12.24)**
Source of information				
Family and friends	12 (75)	4 (25)	1	1
Media	23 (31.5)	50 (68.5)	6.52 (3.89 to 16.03)**	0.60 (0.17 to 2.17)
Health professionals	162 (46.4)	187 (53.6)	3.46 (2.42 to 7.26)**	0.78 (0.18 to 3.48)
Parity				
1	49 (43.8)	63 (56.2)	2.18 (0.99 to 4.75)	0.51 (0.10 to 2.51)
2–4	183 (50.4)	180 (49.6)	1.67 (0.81 to 3.41)	0.61 (0.15 to 2.44)
≥5	22 (62.9)	13 (37.1)	1	1
ANC visits				
1–3	170 (69.1)	76 (30.9)	1	1
≥4	59 (24.9)	178 (75.1)	6.75 (4.53 to 10.07)**	2.10 (1.13 to 3.90)*
ANC counselling				
No	94 (79.0)	25 (21.0)	1	1
Yes	135 (37.1)	229 (62.9)	6.38 (3.91 to 10.41)**	4.33 (1.88 to 9.98)**
Delivery place				
Home	105 (78.4)	29 (21.6)	1	1
Health institution	149 (39.6)	227 (60.4)	5.52 (3.48 to 8.74)**	2.73 (0.49 to 15.34)
PNC counselling				
No	118 (49.0)	123 (51)	1	1
Yes	36 (26.1)	102 (73.9)	2.72 (1.72 to 4.29)**	0.74 (0.38 to 1.45)
Essential newborn care knowledge				
Poor	175 (79.9)	44 (20.1)	1	1
Good	79 (27.1)	212 (72.9)	10.67 (7.02 to 16.24)**	3.91 (2.05 to 7.48)**

COR: crude odds ratio.

*p<0.05, **p<0.001.

The odds of knowledge were 2.1 times greater among mothers who had four or more ANC visits (AOR 2.10 [95% CI 1.13 to 3.90]) compared with mothers who had three or fewer ANC visits. Utilization of counselling by mothers during ANC visits was significantly associated with knowledge about neonatal danger signs (AOR 4.33 [95% CI 1.88 to 9.98]). Mothers who had knowledge about essential newborn care were 3.91 times more likely (AOR 3.91 [95% CI 2.05 to 7.48]) to be knowledgeable about neonatal danger signs.

## Discussion

The knowledge of mothers on neonatal danger signs was found to be 50.2% (95% CI 46.3 to 54.3). Factors that were significantly associated with women's knowledge of neonatal danger signs were the mother's education level, husband's education level, place of residence, number of ANC visits, counselling during ANC and knowledge about essential newborn care.

The knowledge of neonatal danger signs in this study was consistent with studies done in southern Ethiopia,^19^ which was 50.3%, and the Tigray region,^24^ which was 50.6%, but was higher than in studies conducted in Kenya,^25^ eastern Ethiopia^14^ and Gondar, Ethiopia^20^ (15.5%, 9.38% and 18.2%, respectively). This difference might be due to study period differences, differences in the data collection tools, the number of neonatal danger signs included (which was higher in this study) and exposure to ANC and the percentage of institutional deliveries (mothers who delivered at health institutions had better exposure to PNC), which were higher in this study. Previous studies included mothers who gave birth 2 y back, which might lead to mothers’ failure to recall. Knowledge of neonatal danger signs in this study was lower than in studies conducted in Baghdad (81%),^26^ Sri Lanka (80%),^27^ Nigeria (78.7%)^10^ and Wolkite, Ethiopia (68.68%).^11^ This might be due to the involvement of rural women in this study and the study area difference in that this study was a community-based study. In this study, a low level of mothers’ knowledge of neonatal danger signs was observed even though the majority of the women had attended more than three ANC visits. This led to the idea that ANC providers may not have proper resources and facilities to educate mothers about neonatal danger signs. Poor knowledge of mothers on neonatal danger signs will have a negative impact on the Integrated Management of Childhood Illnesses program in Ethiopia,^28^ because the program is based on early identification of newborn danger signs by caregivers and appropriate referral aimed at reducing neonatal mortality.

Neonates who had danger signs were at a higher risk of death than those who did not.^29^ The healthcare-seeking practices of mothers for their newborns by recognizing those serious illnesses is important to avoid delays, which contributes to neonatal mortality. The most frequently mentioned danger signs were fever, vomiting and lethargy, which is consistent with studies conducted in Kenya^25^ and Ethiopia.^19^ This might be because these signs commonly affect the health of neonates and are relatively easily detected by caregivers. However, this is incongruent with studies conducted in Nigeria^10^ and Ethiopia.^9,20^ This might be due to the difference in health extension workers’ counselling in the community and sociocultural variations between the study participants. Overall, this implies that poor recognition of danger signs is the main barrier to seeking care (only 61.2% of mothers in this study perceived the recognized signs as severe).

In this study, mothers with a secondary or higher education level were about 2 times more likely to know about neonatal danger signs compared with mothers with a primary or lower education level. This is nearly consistent with a study conducted in Gondar.^20^ The possible justification could be that educated mothers acquire knowledge about disease and human health through their academic life and education, utilize health services more and are able to read and understand materials related to newborn health.

Similarly, the husband's education level was significantly associated with the mother’s knowledge about neonatal danger signs. The odds of having knowledge about neonatal danger signs was 2 times greater among mothers whose husbands achieved a secondary or higher education level. This is consistent with the studies conducted in Wolkite and Gondar.^11,19^ This could be explained as educated husbands are more informed and help the mother recognizing neonatal danger signs, which might positively affect the knowledge of the mothers.

The study showed that mothers who live in an urban area are 5.83 times more likely to be knowledgeable compared with mothers who live in rural areas. This is congruent with the study conducted in southern Ethiopia^19^ and Woldia,^9^ which found that living in a rural area increases the odds of good knowledge of neonatal danger signs. This might be because mothers who live in urban areas are more likely to seek healthcare and health information from different sources compared with mothers who live in rural areas. This study showed that 82% of home deliveries were conducted in rural areas, and with a reduced chance to contact a health professional. This leads to the idea that mothers who live in rural areas have a tendency to deliver at home and had fewer opportunities to get immediate PNC counselling on neonatal danger signs.

ANC visits were significantly associated with the mother's knowledge about neonatal danger signs. In line with that, mothers who got counselling during ANC follow-ups were about 4 times more knowledgeable as compared with mothers who did not receive counselling during ANC visits. This is consistent with a study conducted in Ghana,^30^ eastern Ethiopia,^14^ Jimma (Ethiopia)^12^ and Gondar.^20^ This might be because ANC follow-ups may increase the chance to obtain more information related to neonatal danger signs from health professionals. This might indicate the need to improve the counselling given to mothers during ANC and PNC, with emphasis on the signs and symptoms of serious newborn illness.

Mothers who had knowledge about essential newborn care had a significant association with knowledge about neonatal danger signs. This is consistent with another study conducted in Ethiopia.^19^ This might be because the recognition of neonatal danger signs is one of the components of essential newborn care. Thus those mothers who have knowledge of essential newborn care practices are more likely to be knowledgeable about neonatal signs of illness. Also, there was a high rate of institutional delivery in this study (73.7%), which provided greater contact with health professionals and counselling on neonatal danger signs at the appropriate time.

## Limitations of the study

The study does not show a cause-and-effect relationship because of the nature of the study design (cross-sectional). It might have been affected by recall bias, as it included mothers who gave birth in the past 6 months. The study was based on reported rather than observed practices.

## Conclusions

The study revealed that the overall level of the mothers’ knowledge about neonatal danger signs was low and care-seeking practices for sick newborns were unsafe. Only half of the mothers achieved the criteria of WHO regarding good knowledge of neonatal danger signs. Mothers’ practices for neonatal danger signs were unsafe, as most mothers took their sick neonates to traditional healers and used home remedies. In general, this study identified that the mother's education level, husband's education level, place of residence, number of ANC visits, counselling during ANC and knowledge about essential newborn care were independent factors associated with the mother's knowledge of neonatal danger signs. Intervention modalities focusing on maternal/parental counselling on the most common symptoms of illness in the neonate, particularly during the ANC/PNC follow-up as well as during institutional delivery, are essential to increase the mothers’ knowledge of the recognition of illness and to improve care-seeking practices. Moreover, healthcare providers need to educate mothers on neonatal danger signs, mainly focusing on the six danger signs (cold to touch, difficulty breathing, umbilical cord redness/pus, convulsion, jaundice and chest retraction) that most mothers did not know well.

## Data Availability

Pertinent data are presented in this article. Additional data can be requested from the corresponding author upon reasonable request.
